# Early biofilm and streamer formation is mediated by wall shear stress and surface wettability: A multifactorial microfluidic study

**DOI:** 10.1002/mbo3.1310

**Published:** 2022-08-16

**Authors:** Alexander L. M. Chun, Ali Mosayyebi, Arthur Butt, Dario Carugo, Maria Salta

**Affiliations:** ^1^ School of Biological Sciences, Faculty of Science and Health University of Portsmouth Portsmouth UK; ^2^ Department of Mechanical Engineering, Faculty of Engineering and Physical Sciences University of Southampton Southampton UK; ^3^ School of Pharmacy & Biomedical Sciences, Faculty of Science and Health University of Portsmouth Portsmouth UK; ^4^ Department of Pharmaceutics, UCL School of Pharmacy University College London London UK; ^5^ Department of Microbial Corrosion and Biofilms Den Helder The Netherlands

**Keywords:** biofilm, biofilm formation, biofouling, microfluidics, wall shear stress, surface wettability, biofilm streamers, *Cobetia marina*, *Pseudomonas aeruginosa*

## Abstract

Biofilms are intricate communities of microorganisms encapsulated within a self‐produced matrix of extra‐polymeric substances (EPS), creating complex three‐dimensional structures allowing for liquid and nutrient transport through them. These aggregations offer constituent microorganisms enhanced protection from environmental stimuli—like fluid flow—and are also associated with higher resistance to antimicrobial compounds, providing a persistent cause of concern in numerous sectors like the marine (biofouling and aquaculture), medical (infections and antimicrobial resistance), dentistry (plaque on teeth), food safety, as well as causing energy loss and corrosion. Recent studies have demonstrated that biofilms interact with microplastics, often influencing their pathway to higher trophic levels. Previous research has shown that initial bacterial attachment is affected by surface properties. Using a microfluidic flow cell, we have investigated the relationship between both wall shear stress (τ_w_) and surface properties (surface wettability) upon biofilm formation of two species (*Cobetia marina* and *Pseudomonas aeruginosa*). We investigated biofilm development on low‐density polyethylene (LDPE) membranes, Permanox® slides, and glass slides, using nucleic acid staining and end‐point confocal laser scanning microscopy. The results show that flow conditions affect biomass, maximum thickness, and surface area of biofilms, with higher τ_w_ (5.6 Pa) resulting in thinner biofilms than lower τ_w_ (0.2 Pa). In addition, we observed differences in biofilm development across the surfaces tested, with LDPE typically demonstrating more overall biofilm in comparison to Permanox® and glass. Moreover, we demonstrate the formation of biofilm streamers under laminar flow conditions within straight micro‐channels.

## Graphical abstract

We used a microfluidic platform to investigate biofilm development by applying four different wall shear stresses simultaneously and tested three different surfaces with varying hydrophobicity. Our key results showed that surface wettability played a role in biofilm formation for *Cobetia marina* and *Pseudomonas aeruginosa* at low shear stress. However, this was minimized as the shear stress increased. Biofilm streamers were observed at the highest shear stress.

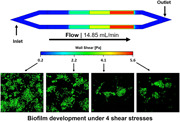

## 1 INTRODUCTION

Biofilms are collections of microorganisms adhered to a surface (living or inanimate), or as flocs, and encapsulated by a network of extra‐polymeric substances (EPS), providing the constituent organisms with enhanced protection from both environmental stressors and antimicrobial substances (Costerton, 1999; H. Flemming & Wingender, 2010). Biofilms are the prominent growth form of bacteria and can be characterized by intricate, three‐dimensional microstructures which allow for liquid through flow and the generation of nutrient gradients (Hans Curt Flemming & Wuertz, 2019; Kolter & Greenberg, 2006).

The detrimental effects of biofilm development are prevalent in a vast range of fields, including industrial, ecological, and medicinal settings (Donlan, 2001; Michael P. Schultz, 2007; Salta, Wharton, Blache, et al., 2013; Vertes et al., 2012). These profound negative consequences can be demonstrated clearly in a range of scenarios and sectors, for example in biofilm‐related infections (medical), food processing, to the maritime sector (H. C. Flemming, 2002; Guaglianone et al., 2010; Høiby et al., 2011). Biofouling is the progressive accumulation of organisms upon submerged surfaces and biofilms, aka microfouling, act as the precursor stage (M. P. Schultz et al., 2011). The incremental build‐up of fouling leads to rapid system clogging, biocorrosion, reduction of operational sensitivity in environmental sensors, and increased rates of hydrodynamic drag on ships (Delauney et al., 2010; Neria‐González et al., 2006; M. P. Schultz et al., 2011). Within medical settings, the presence and persistence of biofilms create a serious concern (Monteiro et al., 2009), for example, implants, such as urinary catheters, arterial stents, artificial joints, and dental implants are highly susceptible to biofilm formation, providing an artificial surface for microorganisms to colonize (Guaglianone et al., 2010; Percival et al., 2017). With the established increased rates of antimicrobial resistance associated with biofilms, indwelling infections are much harder to treat and remove (Vertes et al., 2012). Research in this field has previously focused on improving and modifying the materials used in implants to increase their anti‐biofilm capability, with the incorporation of antimicrobial compounds (Jordan et al., 2015; Von Borowski et al., 2019).

Initial bacterial attachment and subsequent biofilm formation are intrinsically linked with the dynamic conditions of the surrounding environment (Dunsmore et al., 2002; J. Kim et al., 2013; Shumi et al., 2010). Factors such as pH, temperature, hydrodynamic forces, and the properties of the surface the biofilm colonizes dictate both the rate and eventual extent of biofilm formation (Jeong et al., 2014; Karimi et al., 2015; Stewart, 2012). Biofilm development is strongly influenced by the surrounding flow and the associated wall shear stress (τ_w_) levels (Tsagkari & Sloan, 2018). It has been shown that the flow field can impact features such as community composition, physical structures, and growth (Purevdorj et al., 2002; Rupp et al., 2004; Wang et al., 2018). It has also been established that τ_w_ can modulate the growth stages in biofilm development, with an extended immature stage demonstrating a clear adaptation to flow conditions (Rickard et al., 2004; Rochex et al., 2008). In the current research, we include a series of τ_w_ levels that are simultaneously applied to investigate their effect on biofilm early establishment, allowing for detailed and direct comparisons between flow conditions.

The adhesion of bacteria to a surface is the fundamental and primary process in biofilm development and depends upon the surface properties of any substrate being colonized, with attachment relying upon attraction forces between bacterial cells and the surface, described by the extended DLVO theory (named after Boris Derjaguin and Lev Landau, Evert Verwey and Theodoor Overbeek) (Katsikogianni et al., 2004; Tuson & Weibel, 2013). This close relationship between surface properties and bacterial attachment has been targeted within anti‐biofilm research to identify strategies aimed at limiting the extent of biofilm formation (Bohinc et al., 2014). Factors such as surface topography, wettability, and surface energy have been modified, alongside the addition of embedded antimicrobial substances, in successful attempts to reduce rates of bacterial attachment while also producing a surface from which biofilms can be more readily removed (Arpa‐Sancet et al., 2012; Pasmore et al., 2002; Sanchis et al., 2006). Surfaces selected in the current work are characterized by different surface energies, allowing for a comparative quantification of the effect of substrate properties on biofilm growth.

Several biofilm morphologies associated with continued biofilm development have been shown to increase the detrimental impact of biofilms within flow cell environments (Drescher et al., 2013; Marty et al., 2012). Biofilm streamers, which are extensions of the bulk biomass that are suspended in the surrounding flow have gained notoriety, with increasing research aiming to characterize early formation and development due to their established links to higher rates of clogging, increased maintenance costs, decreased flow rates, limits to the efficiency of self‐cleaning systems, and a reduction in an operational lifetime (Drescher et al., 2013; Stoodley et al., 1998). Previous research into the fundamental aspects of streamer formation has concluded that the convergence of flow over biofilm can extend the biomass and cause an elongation of the EPS which trails behind, suspended in the flow (Rusconi et al., 2010). Experimental and mathematical simulations have demonstrated the formation of streamers creates a more streamlined biofilm profile, which reduces the hydrodynamic force the bulk biomass is subject to, resulting in a more resilient biofilm (Taherzadeh et al., 2010).

While previous research has evaluated the role of either fluid shear stress or surface properties on biofilm development, we have utilized a microfluidic‐based platform to investigate these effects simultaneously (B. Li & Logan, 2004; Shumi et al., 2013). Microfluidic devices can be purpose‐built and are entirely customizable to suit experimental designs across a vast range of fields including biotechnology, microbiology, and pharmaceutics (K. P. Kim et al., 2010; Sackmann et al., 2014; Zheng et al., 2016). Flow cells are a class of devices that involve the manipulation of liquids through a defined experimental area, to replicate flow metrics that are relevant to specific applications, i.e. τ_w_ or fluid velocity in physiological systems (X. Li et al., 2012; Nance et al., 2013; Runyon et al., 2008). These small‐scale devices have allowed for detailed examination within biofilm research, on aspects such as bacterial attachment and biofilm development (K. P. Kim et al., 2010; Samarian et al., 2014).

In the current work, three different surfaces were used to test initial bacterial attachment and biofilm development; these substrates were chosen to provide a range of surface properties representative of the wide settings where biofilms are a cause for concern. Low‐Density Polyethylene (LDPE) is a thermoplastic ubiquitously used in numerous applications and products (plastic wraps and bags, squeeze bottles, toys, and gas and water pipes). Plastic and microplastic pollution is a major issue in the marine environment, therefore shedding light on bacterial colonization and biofilm formation on this material is crucial (Kooi et al., 2017; Michels et al., 2018; Rummel et al., 2017). The second surface used was Permanox®, which is an inert surface frequently used for cell attachment and growth. Finally, the third surface used was glass, which is a standardized material used in every laboratory worldwide. Using a range of surfaces with different surface energies, we aimed to identify any difference in biofilm development and, in turn, demonstrate the close relationship with substrate properties. Moreover, the occurrence of streamers in straight channels remains a largely unexplored area, which we further investigate in the current work. Existing research on biofilm development routinely utilizes micro‐channels with complex internal geometries to induce flow disruption over and around biofilms, therefore increasing the prevalence of biofilm streamers (Rusconi et al., 2010, 2011). With a significant increase in clogging associated with biofilm streamers, it is crucial to examine the processes that govern their formation to direct future research aimed at their prevention (Drescher et al., 2013; Stoodley et al., 1998). Furthermore, results from this multi‐parametric experimental investigation could potentially inform the development of predictive computational models that could be employed in the design of effective anti‐biofilm surfaces for a range of applications.

## 2 MATERIALS AND METHODS

### 2.1 Flow cell design and fabrication

The flow cell used within these experiments is a second‐generation device, designed and fabricated in the same way as the precursor model, which is detailed in Salta,  Wharton, Blache, et al. (2013). Briefly, the channel architecture was micro‐milled in a layer of polymethyl methacrylate (PMMA), using a Datron CAT3D‐M6 milling machine (Datron Dynamic, Inc.). A recess was also milled within this layer, and a custom‐built silicone gasket was positioned in the recess to allow for effective sealing between the PMMA layer and the substrate surface. The gasket was fabricated from polydimethylsiloxane (PDMS, Sylgard 184, Dow Corning), using a weight ratio of 10:1 between PDMS monomer and curing agent. The device was designed to investigate the performance of fouling‐control surfaces/coatings under different wall shear stress levels and to create a standardized test for the development of new coatings for the industries that eventually deploy them. While the number of parallel channels has decreased from six to four from the initial design, the original premise of channels with decreasing heights generating a range of wall shear stress levels remains. The internal dimensions of these channels have been altered and optimized to extend the range of τ_w_ generated.

**Figure 1:**
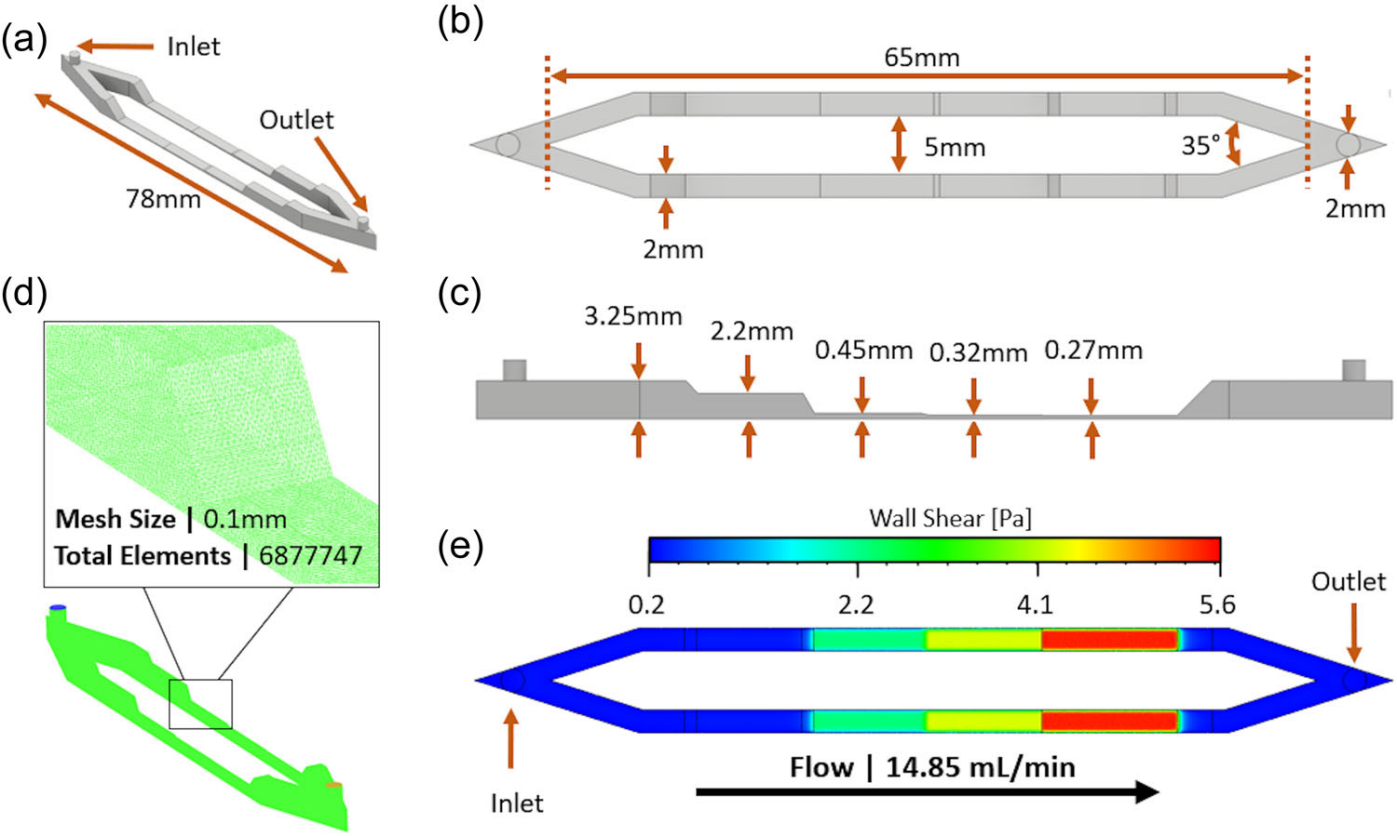
Schematic showing the design elements of the microfluidic flow cell, where (a) the span of the microfluidic channels, (b) top‐down view of the channels, (c) cross‐sectional view of the channel, showing the step‐like progression of the chambers, (d) the mesh size and total elements in the flow cell design, features critical to the CFD calculations, and (e) the wall shear stress over the bottom surface of the chambers as determined from the numerical simulations. CFD, computational fluid dynamic

While the first device had an inlet for each of the six channels, the current device has two inlets each leading into two channels, containing four chambers in each channel, as shown in Figure 1a–c. The τ_w_ values generated within these chambers are relative to those present in tidal, blood flow, or cooling systems generating highly representative results for each of these settings (Cowle et al., 2020; Ku et al., 1985; Manuel et al., 2007). Moreover, while in the original design the channel height decreased in a step‐like fashion, in the present device a more gradual (e.g., tapered) transition between chambers of different heights was established. This was aimed at preventing the onset of vortical flow in these regions of the device, which may potentially act as entrapment sites for flowing bacteria.

The fluid dynamic field within the device was determined from three‐dimensional (3D) computational fluid dynamic (CFD) simulations based on the finite volume method, using ANSYS Fluent (ANSYS, Inc.). The model domain was discretized in 6,877,747 mesh elements, having an edge length of 0.1 mm. The flow field was determined by solving for mass and momentum conservation (i.e., Navier–Stokes equations) assuming that the fluid is incompressible and Newtonian and that the flow is steady and laminar. The volumetric density and dynamic viscosity of the fluid were set to 998 kg/m^3^ and 0.001 Pa·s, respectively. A volumetric flow rate of 14.85 ml/min was imposed at the inlet cross‐section of the device, while atmospheric pressure was set at the outlet. A no‐slip boundary condition was instead imposed on the inner walls. The flow metric of primary interest in this study was the wall shear stress acting over the bottom surface of the device, which was defined as the force per unit area exerted by the moving fluid on the surface, in a direction parallel to the surface itself.

### 2.2 Contact angle measurements

Contact angle (CA) measurements for each of the three test surfaces used in this study were performed to relate surface characteristics to biofilm development (KSV Instruments LTD, CAM101). The static measurements were conducted with ultra‐filtered deionized water and were repeated 10 times for each surface, and the average and standard deviation of the contact angle values were calculated. Images were captured the moment the water droplet touched the surface and were used to calculate the contact angle.

### 2.3 Bacterial attachment and biofilm development assays

The species used in these experiments included *Cobetia marina* ATCC25374, as it has been previously employed as a model species in multiple attachment assays (Mieszkin et al., 2012; Salta, Wharton, Dennington et al., 2013). *C. marina* aliquots were taken from cryopreserved stocks stored at –80°C, plated onto marine agar (BS DifcoTM Marine Agar 2216), and incubated at 25°C. After an initial growth period of 48h, a single colony was used to inoculate Sea Salt Peptone (SSP), made using 35 g/L of Sea salts (S9883, Sigma Aldrich) and 18 g/L of Peptone (LP0037, Oxoid). The liquid culture was incubated at 25°C under agitation at 80 rpm, for over 12 h and bacterial growth was measured using a Synergy H1 microplate reader (BioTek®) with optical density (OD) at λ = 600 nm (OD_600_).

The second species used was *Pseudomonas aeruginosa* ATCC25668; this species has been used in medical biofilm research, with certain strains causing infections (Pasmore et al., 2002; Purevdorj et al., 2002). *P. aeruginosa* was taken from cryopreserved stocks, stored at –80°C, plated onto nutrient agar (Oxoid LP0013), and incubated at 37°C. After 48h, a single colony was transferred to nutrient broth (NB) (Oxoid CM0067) and incubated at 37°C for 12 h. With both bacteria, experiments were initiated once the liquid cultures reached an optical density (OD) at λ = 600 nm (OD_600_) of 0.2.

**Figure 2:**
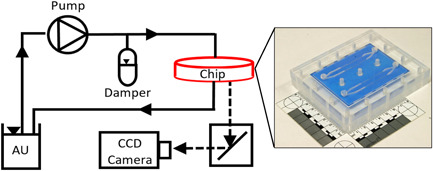
Schematic illustrating the experimental set‐up, showing the constituent parts including an image of the flow cell. Arrows illustrate the direction of flow. Dotted lines indicate the option of adding a CCD camera for real‐time measurements.

A media reservoir containing 500 mL of SSP for *C. marina* or NB for *P. aeruginosa* was connected to a peristaltic pump (Watson‐Marlow series 323S) to generate a continuous flow through the closed set‐up, as shown in Figure 2. The addition of a 0.22 µm sterile filter prevented contamination while allowing aeration of the liquid media. A dampener was used in this setup to create a steady flow, attenuating the pressure wave generated by the pump. All components were connected using silicone rubber tubing (Masterflex, with an internal diameter of 3.1 mm). The flow cell and test surfaces were disinfected using 70% ethanol while all tubing was autoclaved before the start of each experiment. Low‐density polyethylene (LDPE) membrane material was obtained from Fisher Scientific, Nunc™ Permanox® microscope slides from Thermo Scientific, and glass microscope slides from Jaytec Glass Ltd).

An initial 1‐h period was used to condition both the test surfaces and the channels with the appropriate media alone (SSP or NB). A sterile syringe was then used to introduce 5 ml of the desired bacterial culture (OD_600_ = 0.2), after which the bacteria were left to attach for 30 min under static conditions. The flow was then restarted and continued for three and a half hours. The average flow rate achieved through these experiments (14.85 ml/h) was later used in the numerical simulations to accurately quantify the wall shear stress levels on the bottom surface of the device. The flow rate was calculated as described in Table A1. All experiments were conducted at room temperature. Upon completion of the experiment, the nucleic acid stain SYTO™9 (green fluorescent nucleic acid stain that stains all bacteria within the sample; Molecular Probes) was introduced into the flow cell using a sterile syringe, and the set‐up was stored in a dark environment for 25 min at room temperature under static conditions. After this incubation period, any excess stain was washed away by conveying 5 mL of PBS through the flow cell. This procedure did not remove any established biofilm as the applied flow rate for the washing step for both species was the same as the experimental. The experiments were repeated four times per surface, while the multiple channel design shown in Figure 1, allowed for additional four replications within each experiment, enabling robust statistical comparisons between experimental groups (see next section for detailed experimental replication).

### 2.4 Microscopy and image processing

The samples were analyzed using a confocal laser scanning microscope, Zeiss LSM 5 Pascal (Carl Zeiss). For each surface and in each of the four chambers, 20 Z‐stacks were taken (*N* = 20), totaling *N* = 160 stacks per experiment, resulting in a total of *N* = 320 stacks per surface (experiments were repeated four times). All Z‐stack images were recorded at Z‐intervals of 0.5 µm, using a 63X magnification water immersion lens. Images were taken at the center of the channels, at a distance of 0.2 mm away from the sidewalls and the tapered region connecting different chambers in a channel, which can be seen in Figure 1. These measures were taken to avoid recording biomass that may have been attached to the lateral or top surfaces of the channel and not the surfaces being tested. The light exposure for SYTO9 was at λ_EX_ = 488 nm, and the emission was collected between λ_EX_ = 500–600 nm. Volocity software (PerkinElmer, Inc.) was used to improve the quality and resolution of 3D image data sets.

The Z‐stacks were processed using COMSTAT2, a plugin within the image processing software ImageJ (MacBiophotonics ImageJ) (Heydorn et al., 2000). A fixed threshold value and connected volume filtration were used throughout all image processing and analysis. Using COMSTAT2, the biofilm biomass (µm^3^/µm^2^), maximum thickness (µm), area occupied in layers, and surface area (µm^2^) were determined. Biomass is calculated as the volume of all voxels, above a threshold calculated as per Otsu's method (Otsu, 1979), that contains biomass divided by the total image area. Maximum thickness is defined by the highest point of the biofilm relevant to the substratum. The area occupied in layers is the biomass recorded within each slice of a Z‐stack, and the surface area accounts for the area occupied by the biofilm.

### 2.5 Statistical analysis

Data processed with COMSTAT2 (biomass, surface area, and maximum thickness) were analyzed for statistical differences using IBM SPSS statistics 24. To determine the homogeneity of variances, Levene's test was used. In cases where the data failed to meet the homogeneity of variances, a Kruskal–Wallis test was used; otherwise, a one‐way analysis of variance (ANOVA) was used. In cases with multiple parameters, a multivariate analysis of variance (MANOVA) was applied. All conclusions were based on a 95% confidence level Regression analysis (linear fit) was performed to determine significant differences among the different shears and scatterplots were generated using OriginPro 2020b.

## 3 RESULTS AND DISCUSSION

### 3.1 Flow characterization

The computer‐aided design elements of the flow cell, shown in Figure 1d,e, were used in CFD simulations to predict the wall shear stress field acting over the biofilm surface in the experiments. The flow regime generated in our experiments was characterized as steady and laminar throughout the whole device. As shown in Table 1, the τ_w_ generated from a volumetric flow rate of 14.85 ml/min ranged from 0.1 Pa to 5.6 Pa along the flow channel. Given that the fluid velocity has a parabolic profile, the wall shear stress in close proximity to the side walls of the channel is lower when compared to the central region. The reported wall shear stress values have thus been determined at a distance >0.2 mm away from the lateral walls, where the wall shear stress is substantially uniform. Notably, the wall shear stress values achieved are within the range biofilms experience in dental environments and physiological or pathological arterial blood flow (Guaglianone et al., 2010; Ku et al., 1985; Nance et al., 2013; Samarian et al., 2014). It should also be noted that the overall residence time of flowing cells within the device is <1 s.

**Table 1:** Values of wall shear stress, mean velocity, and Reynolds number in the microfluidic flow chambers

τ_w_ (Pa)	Velocity (ms^−1^)	*Re*
0.2	0.0281	65.9
2.2	0.138	113
4.1	0.193	119
5.6	0.229	122

### 3.2 Surface characterization

The surface wettability of all three surfaces tested in the current experiments was determined by measuring the corresponding contact angle. Each surface demonstrated different degrees of hydrophobicity, with Permanox® being the most hydrophobic (i.e., having a CA > 90°) and glass being hydrophilic (Samuel et al., 2011). The observed results are consistent with the ranges reported in existing literature for all three surfaces (Deng et al., 2002; B. Li & Logan, 2004; Sanchis et al., 2006; Trentin et al., 2015). Specifically, the following CA values were found for each surface: (a) LDPE = 86.4° (±7.3°), (b) Permanox® = 93.6° (±6.1°), and (c) glass = 25.9° (±10.4°). Bacterial attachment relies upon cell‐to‐surface interactions and as such surface properties are intrinsically linked to later biofilm development (Hori & Matsumoto, 2010); it has also been shown that increasing the surface roughness of glass can directly increase the rate of bacterial adhesion (Bohinc et al., 2014).

### 3.3 Biofilm responses

#### 3.3.1 Biofilm characteristics with increasing wall shear stress

**Figure 3:**
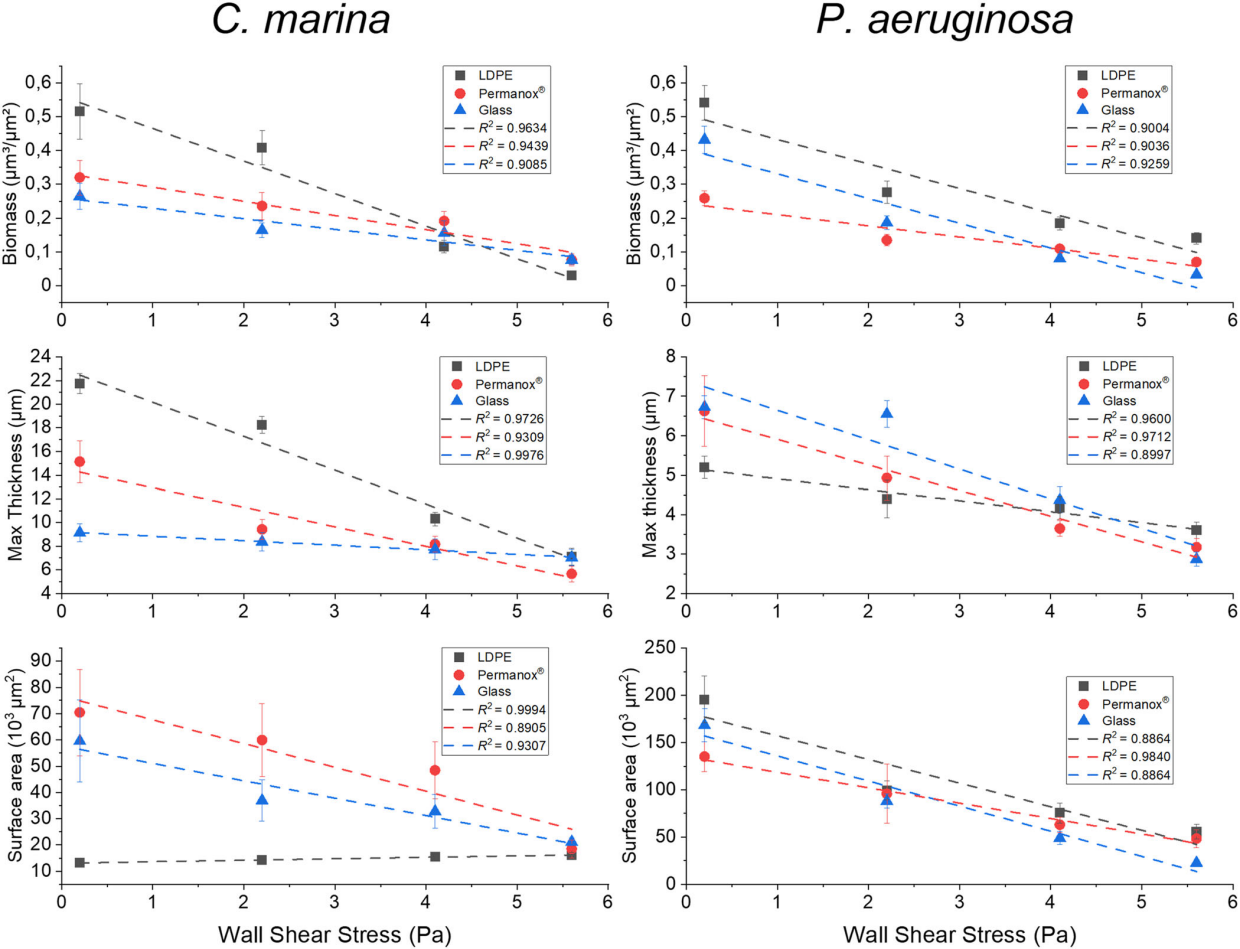
Influence of shear stress on *Cobetia marina* on the left and *Pseudomonas aeruginosa* on the right‐hand side when exposed to different surfaces. Results collected from each test surface are compiled above including the *R*² value for the linear fit, in the order of LDPE, Permanox® and Glass, respectively. Error bars ± SE. Note: the associated statistical analysis can be found in Tables A2, A3, A4, A5. SE, standard error

Several parameters were measured from the biofilm images acquired under each of the wall shear stress values, for both bacterial species used, and data are presented in the form of regression charts in Figure 3. The overriding trend is that with an increase in wall shear stress the biomass, maximum thickness, and surface area decrease across all three surfaces investigated, regardless of species.

In multiple cases for biomass, maximum thickness, and surface area, significant differences can be found when τ_w_ increases by an order of magnitude of two (i.e., 0.2 Pa is significantly different from 4.1 Pa, but not 2.2 Pa), as shown in Figure 3 and Tables A2, A3. These observations suggest that the end‐point biofilm formation metrics are inversely proportional to wall shear stress. Such trends support previous findings suggesting that changing shear stress alters formation patterns of bacterial biofilms, with higher τ_w_ restricting formation more than lower τ_w_ (Liu & Tay, 2002; Paul et al., 2012). For *C. marina* biomass on LDPE, a significant difference was observed between the lowest and highest τ_w_, with lower τ_w_ supporting greater biomass (Table A2, A3). For biomass on both Permanox® and glass, significant differences were found when the highest τ_w_ (5.6 Pa) was compared to the lowest two values (0.2 Pa, 2.2 Pa). Glass showed no significance for maximum thickness between any τ_w_ values investigated, although significantly higher biomass was recorded under lower τ_w_ (0.2 Pa vs. 5.6 Pa [P_KW_ = «0.001], 2.2 Pa vs. 5.6 Pa [P_KW_ = 0.026]). Overall, the glass produced comparatively thinner biofilms regardless of the applied wall shear stress value, highlighting that the hydrophilic nature of the surface limited biofilm development. While differences were recorded for biomass and maximum thickness, no significant differences were observed for the surface area between any wall shear stress on all three surfaces for *C. marina* (Table A2).

For *P. aeruginosa* biomass, significant differences were also (like with the marine species) recorded when τ_w_ increased by an order of magnitude of two on all three surfaces (0.2 Pa is significantly different from 4.1 Pa and 5.6 Pa, but not 2.2 Pa), showing again that increasing τ_w_ affects biofilm development incrementally. Where maximum thickness is concerned, LDPE and Permanox® only showed significance between the lowest and highest τ_w_ (LDPE 0.2 Pa vs. 5.6 Pa [*p* = 0.005], Permanox® 0.2 Pa vs. 5.6 Pa [P_KW_ = «0.001]). Conversely, a wider range of biofilm parameters was impacted on glass in relation to wall shear stress, with only 0.2 Pa and 2.2 Pa not showing significant differences. As shown in Figure 3, experimental data for both species and all surfaces within these experiments were linearly interpolated, and the corresponding coefficient of determination (*R* ^2^) values were calculated. There is a good fit with the linear regression model (*R*² in the range 0.88–0.99), indicating that the reduction shown in overall biofilm formation can be explained by the corresponding wall shear stress increase. It may have been expected that biofilms would have detached predominately above a critical value of wall shear stress, resulting in a nonlinear relationship between biofilm metrics and wall shear stress. This was not observed in the present study and may be potentially attributed to the fact that different regions of the biofilm interact differently with the imposed fluid flow, that is, due to spatial differences in biofilm morphology and/or the effect of these on local flow patterns.

These results quantitatively validate and further extend previous observations made using microfluidic flow cells (Salta, Capretto, Carugo, et al., 2013). The linear regression functions also allow for the prediction of biofilm characteristics that could be expected under different wall shear stress levels on each surface. The current results add to the increasing body of research that links higher τ_w_ with reduced biofilm development (Conrad & Poling‐Skutvik, 2018; Dunsmore et al., 2002; J. Kim et al., 2013; Paul et al., 2012; Wang et al., 2018). Therefore, the causal relationship between τ_w_ and biofilm formation is clear.

#### 3.3.2 Biofilm development is dependent upon the surface type

**Figure 4:**
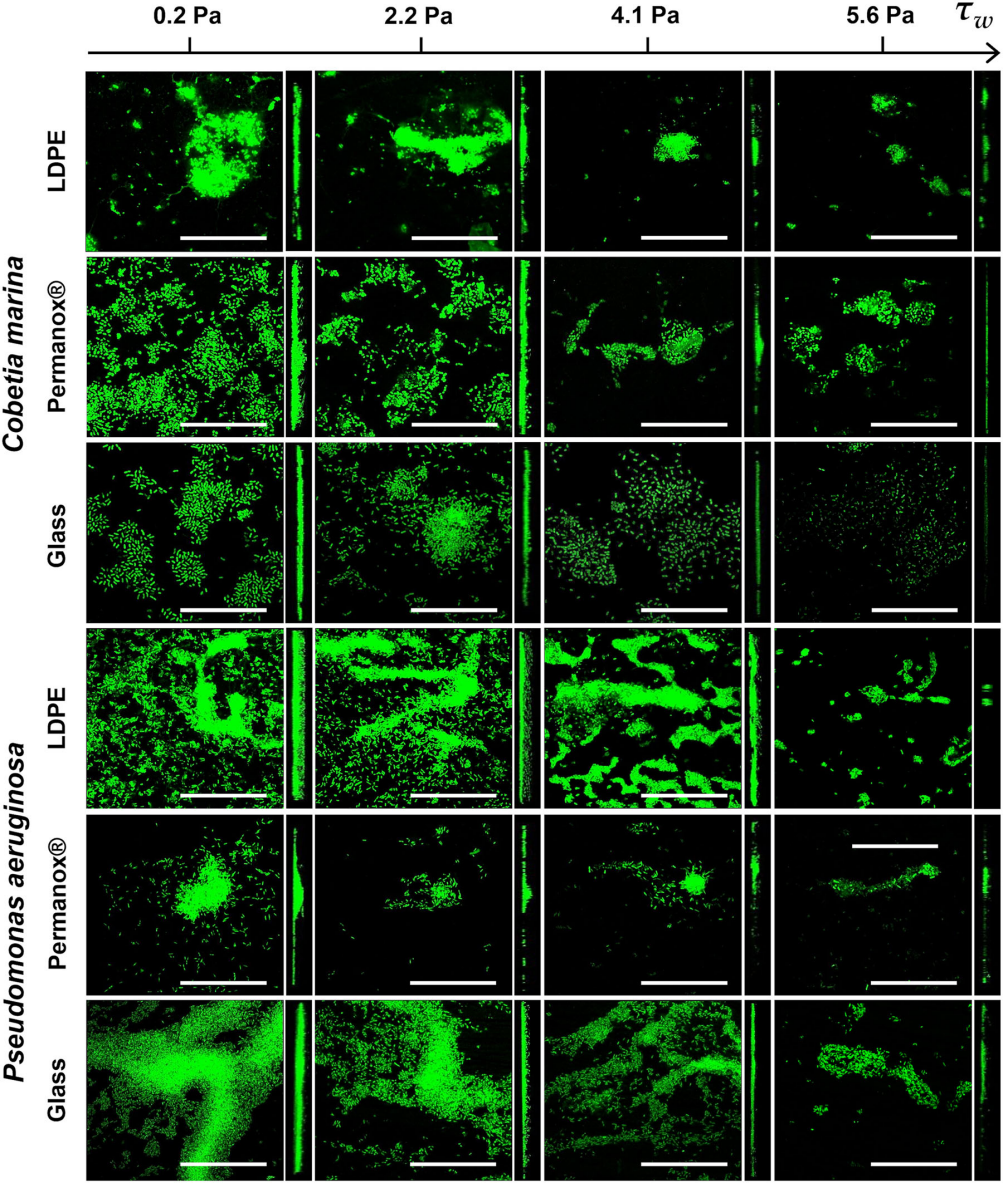
Confocal laser scanning microscopy (CLSM) images of *Cobetia marina* and *Pseudomonas aeruginosa* biofilm on low‐density polyethylene (LDPE), Permanox®, and glass showing both the XY and XZ planes. In all images, the flow was oriented from left to right, with scale bars of 50 µm. These images demonstrate that overall biomass and biofilm thickness decrease as the wall shear stress levels increase.

Biofilm formation was impacted by surface type, as shown in Figure 3; these findings are consistent with the current understanding that surface wettability plays an intrinsic role in biofilm formation and development (Pasmore et al., 2002; Michael P. Schultz, 2007; Tuson & Weibel, 2013; Zheng et al., 2021). This variation between surfaces is associated with surface properties and, by extension, the way cells interact with the surfaces themselves (Katsikogianni et al., 2004; Tuson & Weibel, 2013). To evaluate differences in biofilm formation, the results of each surface at the four τ_w_ values were compared (e.g., at 0.2 Pa: LDPE vs. Permanox® vs. Glass), as shown in Tables A4 and A5. For *C. marina* biofilm biomass, the only significant difference was observed between LDPE and glass at the lowest τ_w_, while in the case of Permanox® there was no significant difference between any τ_w_ value nor when compared to other surface types. Such an observation shows that wall shear stress has a dominant effect on biomass, reducing the intrinsic differences in surface characteristics. This was also observed by Schwarze et al. (2020) who found that although *C. marina* cells attached more on hydrophobic surfaces (in comparison to hydrophilic ones) when shear was introduced (0.45 Pa), this effect was reduced. In terms of maximum thickness, however, the type of surface had a more profound effect. *C. marina* biofilms showed thicker structures at the lower τ_w_ on LDPE followed by Permanox®, while the thinnest biofilms formed on glass (Figures 3 and 4 and Table A4). Once again, as τ_w_ increased, the surface properties had a reduced effect as wall shear stress became the dominant governing factor.

**Figure A1:**
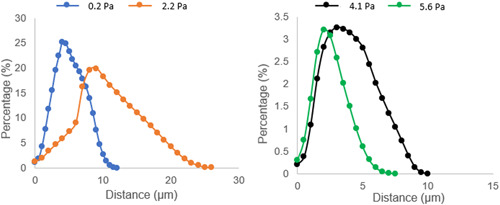
Vertical profiles for *Cobetia marina* biofilm on LDPE showing the percentage coverage in each slice of the Z‐stacks. LDPE, low‐density polyethylene

**Figure A2:**
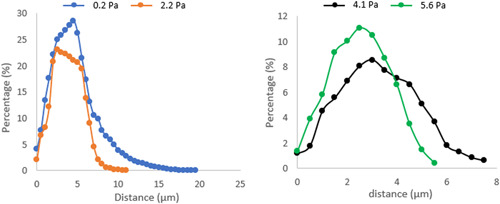
Vertical profiles for *Cobetia marina* biofilm on Permanox® showing the percentage coverage in each slice of the Z‐stacks.

**Figure A3:**
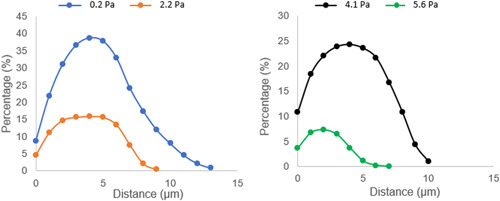
Vertical profiles for *Cobetia marina* biofilm on Glass showing the percentage coverage in each slice of the Z‐stacks and distance from the surface.

**Figure A4:**
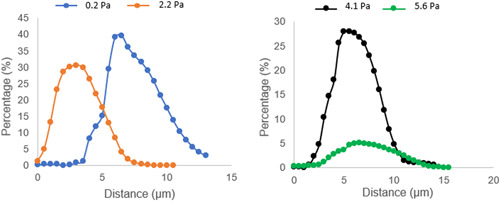
Vertical profiles for *Pseudomonas aeruginosa* biofilm on LDPE showing the percentage coverage in each slice of the Z‐stacks and the distance from the surface. LDPE, low‐density polyethylene

**Figure A5:**
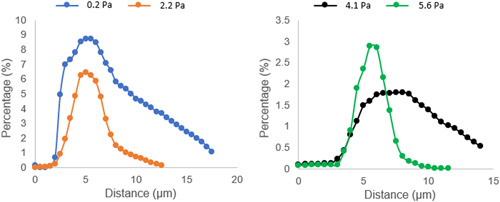
Vertical profiles for *Pseudomonas aeruginosa* biofilm on Permanox® showing the percentage coverage in each slice of the Z‐stacks and the distance from the surface.

**Figure A6:**
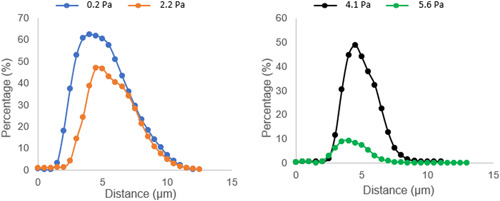
Vertical profiles for *Pseudomonas aeruginosa* biofilm on Glass showing the percentage coverage in each slice of the Z‐stacks and the distance from the surface.

The results for *P. aeruginosa* displayed a less varied biofilm colony distribution than the marine species, where differences between surfaces became less evident. Under all four wall shear stress values, significant differences were found between the two hydrophobic surfaces for biomass, with LDPE showing higher biomass in all cases (LDPE vs. Permanox®, *p* < 0.001). As the τ_w_ increased, the underlying effect of surface properties upon resulting biomass became evident in the case of the hydrophilic glass. Biofilm development on glass resulted in the sharpest decline with increasing wall shear stress (Figures 3 and 4), illustrating that both shear forces and surface properties affect biofilm development. For maximum thickness, the only significant difference between surfaces is found between LDPE and glass, at τ_w_ of 0.2 Pa, 2.2 Pa, and 5.6 Pa (Table A5). There were some clear differences between the two species selected, since *P. aeruginosa* formed thinner biofilms on LDPE than on glass, while the opposite was found for *C. marina* (LDPE showing thicker biofilms than on glass). Surface roughness and stiffness appear to influence *P. aeruginosa* adhesion and c‐di‐GMP production (Zheng et al., 2021). For instance, *P. aeruginosa* illustrated a reduced adhesion on a stainless steel rough surface (Ra: 172.5 nm) when compared with a polished one (Ra: 84.4–45.2 nm); while it has been illustrated that softer material promotes c‐di‐GMP expression and bacterial adhesion (Song et al., 2015). Material stiffness, as an influencing factor towards bacterial adhesion, is still being explored as thoroughly reviewed by Zheng et al. (2021). Although we did not measure the surface roughness and stiffness of our test surfaces, we cannot exclude these factors did not influence the observed differences in biofilm development between the two species. Also, the introduction of flow could exert additional changes in surface properties, and further studies could explore this. Concerning the biofilm surface area, significant differences were found at the highest τ_w_ between glass and both LDPE and Permanox®, with glass displaying the smallest surface area by comparison (LDPE vs Glass [P_KW_ = 0.001], Permanox® vs. Glass [P_KW_ = 0.037]). As shown in Figure 4 (and Figures A1, A2, A3, A4, A5, A6), at the lower τ_w_ (0.2, 2.2 Pa) both species generated comparatively uniform, thicker biofilms covering a relatively larger area of the channel. As the τ_w_ increased, the biomass decreased, promoting more dispersed and overall smaller clusters, as previously observed (Salta, Capretto, Carugo, et al., 2013). At the higher τ_w_ levels (4.1 Pa, 5.6 Pa), the biofilm showed a smaller vertical profile, indicating that the increased wall shear stress limited the vertical development of biofilms (Figures A4, A5, A6). A recent study illustrated that in biofilm formation under temperatures lower than 25°C, the level of intracellular 3',5'‐cyclic diguanylate (c‐di‐GMP), which controls biofilm formation and total exopolysaccharide production, rapidly increases, resulting in more and better‐structured biofilms (Kim et al., 2020). At this point, it should be noted that *P. aeruginosa* biofilm morphology may have also been impacted by the temperature of the experimental setup, which has not been cardinal for this species. And although a temperature effect on biofilm formation cannot be excluded, our experimental duration has been significantly shorter (a total of 4 h) when compared to the study by Kim et al. (2020) that run their experiments for a total of 6 days.

For *C. marina*, biofilm reduction on glass for any parameter is much more gradual when compared to the more hydrophobic LDPE or Permanox®. When results for *P. aeruginosa* on glass are considered, a clear difference from *C. marina* can be noted, with a greater decline of recorded biofilm. Existing research into biofilm development under flow is typically performed on glass surfaces where some exemptions include modified glass and metallic surfaces (Bohinc et al., 2014; Oder et al., 2018). Some studies have also looked at biofilm growth on plastic surfaces targeting water distribution systems, therefore exploring different flow and shear stress regimes (not within microfluidic devices) (Cowle et al., 2020; Manuel et al., 2007). Previous research has compared the attachment of a marine *Pseudomonas* sp. upon a range of surfaces, including polyethylene and glass, and recorded higher attachment to hydrophobic surfaces than hydrophilic (Fletcher & Loeb, 1979). The majority of bacteria genera have a negative net charge, typically defined by zeta potential measurements (Katsikogianni & Missirlis, 2010; Renner & Weibel, 2011); it is well established that electrostatic forces are key in determining bacterial cell attachment to a surface which is also expected to be charged. This interaction can be influenced by the medium's ionic strength, while small molecules, proteins, and ions can alter the surface chemistry and charge via diffusion and mass transport (Renner & Weibel, 2011). In the current study, the surface charge of our test material was not assessed, however, it has been shown that high shear stress can impact the expected surface/bacterial interactions that are based on colloid theories (e.g., DLVO and extended DLVO) and macromolecule binding considerations. Katsikogianni and Missirlis (2010) revealed that simulated hemodynamic shear conditions identified limitations to the colloidal theories and that shear does not allow for direct and exact evaluation of the macromolecular interactions between bacteria and NH2‐terminated surfaces. Here we have demonstrated direct comparisons of three surfaces with different properties that can be used for a variety of applications. With biofilms being ubiquitous in nature, the range of surfaces utilized in the current experiments created an insight into the role played by surface wettability in biofilm formation that can be applied to future investigations, especially addressing the issue of microbial colonization on plastics and microplastics in the environment.

### 3.4 Observation of streamers

**Figure 5:**
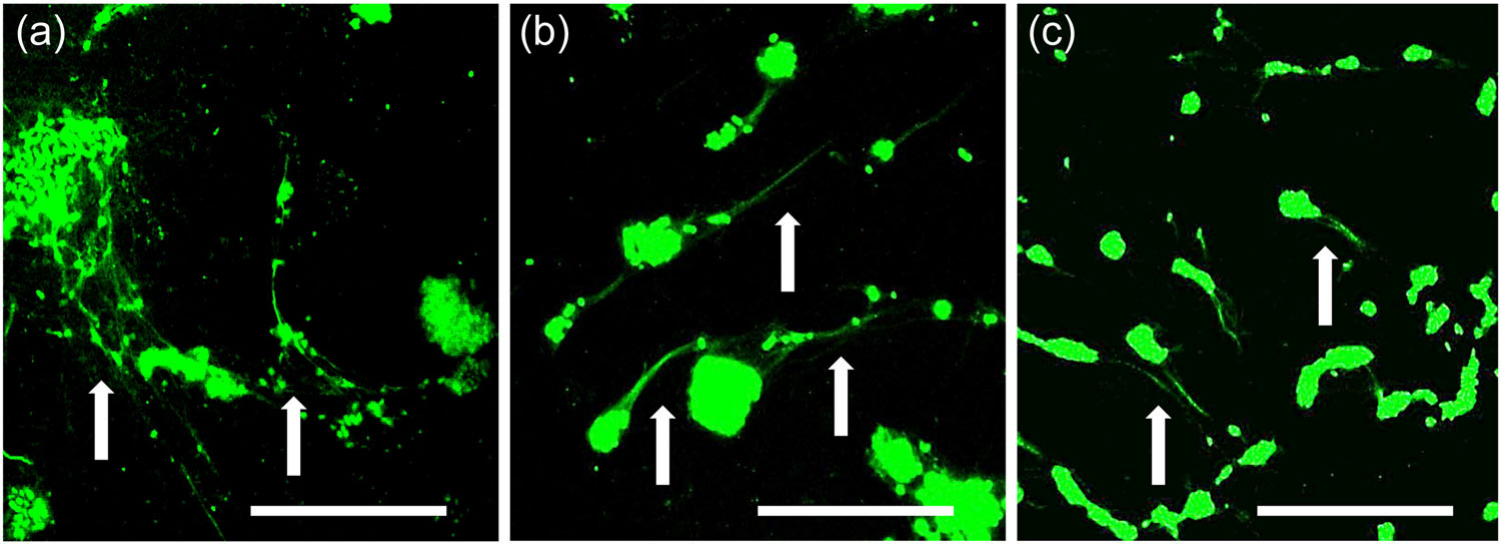
Biofilm streamers recorded under the higher wall shear stress level of 5.6 Pa. Endpoint images of (a) *Cobetia marina* on Permanox®, (b) *Cobetia marina* on glass, and (c) *Pseudomonas aeruginosa* on the glass; streamers indicated by arrows. Images were captured using a 63X objective lens; scale bars are 50 µm. Flow is oriented from left to right in all images. Note: These images were taken under static conditions.

Biofilm streamers of both species were observed upon all surfaces, but exclusively under the highest wall shear stress value (5.6 Pa). These features were observed as small extensions of biofilm clusters, oriented in a similar direction as the flow, as can be seen in Figure 5 (and Figures A1 and A2). The form of the observed streamers varied depending on surface type, with LDPE showing a net‐like structure, while Permanox® and glass showed streamlined streamers. As previously established, these experiments operated under uniform laminar flow within straight channels. This is the first study reporting on such biofilm features under these experimental conditions (i.e., timespan, flow cell geometry, wall shear stress, and range of surfaces). With these experiments, we have shown replication of biofilm streamers across all three surfaces and from both bacteria used. Future related work should investigate such serendipitous results in greater detail and length, including at greater temporal resolution.

Due to the comparatively short run‐time of these experiments, streamers observed here are immature and still in the early stages of development. To date, there have been several studies investigating the formation and the resulting development of such growth features, by performing experiments over extended periods (Persat et al., 2015). Research like Rusconi et al. (2010), who created zig‐zag‐shaped experimental designs specifically to investigate the formation and development of streamers, utilize in situ microscopy to provide continuous data throughout each experiment. They were able to hypothesize that within the flow cell used, the flow at the corners within their microfluidic device creates precursor threads which are then stretched further until the streamers later connect to the next corner. The researchers also suggested that the accumulation of polymeric substances on the channel walls at the corners promotes the formation of these precursor threads. They also noted that streamers begin as pure EPS alone, making early visualization exceedingly difficult. In a follow‐up study, the same research group further investigated the relationship between secondary flow patterns and streamer formation, finding that sharper angles promoted the formation of more elongated and thicker streamers than comparatively shallower angles (Rusconi et al., 2011). These observations do not explain the occurrence of streamers within our research, as the flow cell lacks any complex internal geometric features, such as sharp corners or curved channels, meaning that the observation of streamers under such conditions is unprecedented.

As streamers were only observed under the highest τ_w_ levels, irrespective of the species or surfaces concerned, it can be suggested that the occurrence of streamers is linked to the flow conditions. While this is the first time biofilm streamers have been recorded under a laminar flow regime within straight channels, this relationship between biofilm streamers and flow conditions has been previously established (Conrad & Poling‐Skutvik, 2018; Rusconi et al., 2011; Stoodley et al., 1999). Even though it has been demonstrated that confinement in microfluidic flow cells can affect both biofilm morphology and flow conditions (Drescher et al., 2013; J. Kim et al., 2013; Kumar et al., 2013), the comparatively short run time of the experiments in the current work means that the effects of confinement on streamer formation can be considered negligible. Interestingly, a recent study by Zhang et al. (2017) found that at shear stresses exceeding 200 Pa, up to 25% of *P. aeruginosa* cells adhered tenaciously on a range of tested surfaces even at shear stresses as high as 2000 Pa. It was shown that this subpopulation of *P. aeruginosa* resistant cells was selected by flow, creating strong shear flow persister (SSP) cells. Next to this, Zhang et al. (2017) found that their results indicated the SSP cells can readily form on both hydrophobic (PTFE) and hydrophilic surfaces (clean glass), suggesting the wettability of surface does not have an impact on SSP formation. In our study, streamer formation was also present on all surfaces at the highest τ_w_ regardless of species and surface type. Therefore, it would be very interesting to explore the possibility of a shear‐selective presence of SSP cells with increasing shear stress (as supported by our flow cell design) and a potential for streamer formation by SSP cells. In addition, a study by Rodesney et al. (2017) revealed an increase of c‐di‐GMP with shear for *P. aeruginosa*, therefore it would be interesting to explore the role of this intracellular secondary messenger in streamer formation under shear stress conditions.

## 4 SUMMARY AND CONSIDERATIONS FOR FUTURE WORK

In this study, we have demonstrated that higher wall shear stress levels produce overall thinner biofilms than lower wall shear stress; these findings are consistent with previous research showing that increased shear reduced biofilm thickness (Liu & Tay, 2002; Paul et al., 2012). We have created flow dynamic conditions characterized by a range of wall shear stress levels (0.2–5.6 Pa); varying the inlet flow rate will allow for the recreation of an even wider range of flow conditions which will ultimately relate to a broader field of applications (Samarian et al., 2014). An increased range of wall shear stress values will also allow for a comprehensive analysis of the relationship between shear stress and biofilm development, creating a unique database that could be applicable to an extensive range of settings.

We have shown that the developed flow cell enables high throughput evaluation of different surface types and that the surface properties play a significant role in biofilm development. Therefore, future experiments may investigate a wider range of surface types, relevant to a spectrum of different applications (e.g., other polymers, coatings, and metals). Moreover, alongside contact angle calculations, surface roughness should be evaluated as it can affect bacterial cell attachment (Gharechahi et al., 2012; Song et al., 2015). This is also evident from the current study; for instance, Permanox® and LDPE were comparably hydrophobic, but LDPE supported larger biofilm development. The combinatorial approach to different surfaces and shear stresses provided insights into the prediction of biofilm characteristics that could be expected under different wall shear stress levels on each surface. This can be further developed with the inclusion of an even wider experimental matrix serving different applications and biofilm communities in the environment (e.g., biofilm dynamics on plastics in the aquatic environment) eventually leading to the development of predictive computational models.

Future work should further investigate both the evident formation response to wall shear stress and the formation of streamers under laminar flow in straight micro‐channels, using tailored image acquisition techniques. As streamers begin in the form of nearly pure EPS structures, the inclusion of a fluorescent stain that selectively stains for EPS would allow for targeted and quantitative analysis of any observed streamers in such relatively short‐term experiments (Jeong et al., 2014). The inclusion of modified bacterial strains may also highlight some of the key factors governing formation responses exhibited through experimental investigations (Drescher et al., 2013). While experiments in this study concern mono‐species biofilms, the dominant form of wild‐type biofilms is often multi‐species (Elias & Banin, 2012; Rendueles & Ghigo, 2012; Rickard et al., 2003). The complex composition of such biofilms critically influences their form, with constituent species playing specific roles within (Lee et al., 2014; Yang et al., 2011). The flow cell used in the current work could be used to investigate the effect of flow upon multi‐species biofilms, later drawing direct comparisons against experiments using individual species. The incorporation of molecular analysis techniques could also address whether gene expression differs from multi‐ to mono‐species, aiming to define any potential mechanism that increases resistance to shear stress. Existing research has shown that wall shear stress also influences biofilm community composition, typically reducing overall diversity (Rickard et al., 2004; Rochex et al., 2008). It has been shown that shear stress maintains the biofilm in a young state which is characterized by lower diversity (Rochex et al., 2008). Future multi‐species biofilm experiments could utilize a broad range of communities under various shear stress levels to investigate the specifics of such a response to flow, aiming to determine factors such as a threshold shear stress value where this change in communities begins. When both the current and published research are concerned, it becomes clear that bacterial attachment is intrinsically linked to both surface properties and the surrounding conditions. Future efforts should consider such conclusions paramount when designing new experimental matrices to investigate bacterial attachment and resulting biofilm formation.

## AUTHOR CONTRIBUTIONS

**Alexander Lai Man Chun**: Data curation—equal, investigation–equal, writing—original draft–lead. **Ali Mosayyebi**: Methodology—supporting, writing—review & editing—supporting. **Arthur Butt**: Resources—equal, writing—review & editing—supporting. **Dario Carugo**: Methodology—equal, resources—equal, writing—original draft‐supporting, writing—review & editing—supporting. **Maria Salta**: Conceptualization—lead, funding acquisition—equal, resources–equal, supervision—lead, writing—original draft‐supporting, writing—review & editing—equal.

## CONFLICTS OF INTEREST

None declared.

## ETHICS STATEMENT

None required.

## Figures and Tables

**Figure A7:**
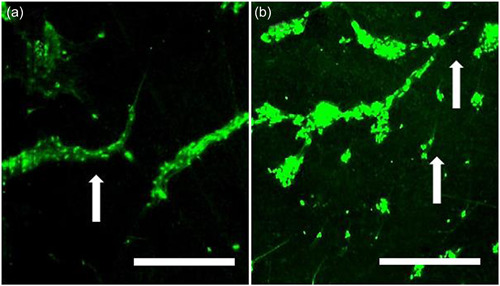
Streamer images recorded on LDPE using confocal laser scanning microscopy with a 63x objective lens, scale bars are 50 *µ*m. Flow is orientated from left to right. (a) shows *Cobetia marina* (b) shows *Pseudomonas aeruginosa*. LDPE, low‐density polyethylene

## Data Availability

All data generated or analyzed during this study are included in this published article.

## APPENDIX A

See Tables A1, A2, A3, A4, A5 and Figures A1, A2, A3, A4, A5, A6, A7.

**Table A1:** The flow rate was calculated at the end of each experiment with the average time (N=5) taken to fill a 10 ml vessel recorded and used to determine the overall millilitre per minute

Average Time to fill 10ml (s)	STDEV (s)	ml/s	ml/m
40.4	1.81	0.2475	14.85

*Note*: This calculation was completed a total of six times with an average flow rate of 14.85 ml/m, the figure used to determine the shear stresses achieved in our experiments.

**Table A2:** *p* values from the statistical analysis comparing *Cobetia marina* results from each shear stress for all three surfaces

Surface	Shear	Biomass	Maximum thickness	Surface area
**LDPE**	5.6 Pa–4.1 Pa	P_KW_ = 0.051	*p* = 0.015	P = 1.000
	5.6 Pa–2.2 Pa	P_KW_ = 0.000	*p* = 0.000	*p* = 0.947
	5.6 Pa–0.2 Pa	P_KW_ = 0.000	*p* = 0.015	*p* = 0.183
	4.1 Pa–2.2 Pa	P_KW_ = 0.005	*p* = 0.000	P = 1.000
	4.1 Pa–0.2 Pa	P_KW_ = 0.001	*p* = 0.000	*p* = 0.667
	2.2 Pa–0.2 Pa	P_KW_ = 1.000	*p* = 0.006	P = 1.000
**Permanox®**	5.6 Pa–4.1 Pa	*p* = 0.148	P_KW_ = 0.172	*p* = 0.141
	5.6 Pa–2.2 Pa	*p* = 0.014	P_KW_ = 0.018	*p* = 0.141
	5.6 Pa–0.2 Pa	*p* = 0.000	P_KW_ = 0.000	*p* = 0.141
	4.1 Pa–2.2 Pa	P = 1.000	P_KW_ = 1.000	*p* = 0.141
	4.1 Pa–0.2 Pa	*p* = 0.091	P_KW_ = 0.018	*p* = 0.141
	2.2 Pa–0.2 Pa	*p* = 0.640	P_KW_ = 0.175	*p* = 0.141
**Glass**	5.6 Pa–4.1 Pa	P_KW_ = 0.360	P = 1.000	P_KW_ = 0.317
	5.6 Pa–2.2 Pa	P_KW_ = 0.026	P = 1.000	P_KW_ = 0.317
	5.6 Pa–0.2 Pa	P_KW_ = 0.000	*p* = 0.349	P_KW_ = 0.317
	4.1 Pa–2.2 Pa	P_KW_ = 1.000	P = 1.000	P_KW_ = 0.317
	4.1 Pa–0.2 Pa	P_KW_ = 0.053	P = 1.000	P_KW_ = 0.317
	2.2 Pa–0.2 Pa	P_KW_ = 0.594	P = 1.000	P_KW_ = 0.317

*Note*: P = N indicates a result obtained using an ANOVA, P_KW_ = N indicates a result obtained using a Kruskal–Wallis test.

Abbreviations: ANOVA, analysis of variance; LDPE, low‐density polyethylene.

**Table A3:** *p* values from the statistical analysis comparing *Pseudomonas aeruginosa* results from each shear stress for all three surfaces

Surface	Shear (Pa)	Biomass	Maximum thickness	Surface area
**LDPE**	5.6 Pa–4.1 Pa	P_KW_ = 1.000	P = 1.000	P_KW_ = 1.000
	5.6 Pa–2.2 Pa	P_KW_ = 0.019	*p* = 0.517	P_KW_ = 0.048
	5.6 Pa–0.2 Pa	P_KW_ = 0.000	*p* = 0.005	P_KW_ = 0.000
	4.1 Pa–2.2 Pa	P_KW_ = 0.412	P = 1.000	P_KW_ = 1.000
	4.1 Pa–0.2 Pa	P_KW_ = 0.000	*p* = 0.173	P_KW_ = 0.002
	2.2 Pa–0.2 Pa	P_KW_ = 0.017	*p* = 0.517	P_KW_ = 0.148
**Permanox®**	5.6 Pa–4.1 Pa	*p* = 0.509	P_KW_ = 1.000	P = 1.000
	5.6 Pa–2.2 Pa	*p* = 0.028	P_KW_ = 0.061	*p* = 0.440
	5.6 Pa–0.2 Pa	*p* = 0.000	P_KW_ = 0.000	*p* = 0.008
	4.1 Pa–2.2 Pa	P = 1.000	P_KW_ = 1.000	P = 1.000
	4.1 Pa–0.2 Pa	*p* = 0.000	P_KW_ = 0.005	*p* = 0.043
	2.2 Pa–0.2 Pa	*p* = 0.000	P_KW_ = 0.302	*p* = 0.818
**Glass**	5.6 Pa–4.1 Pa	P_KW_ = 0.151	*p* = 0.003	P_KW_ = 0.105
	5.6 Pa–2.2 Pa	P_KW_ = 0.000	*p* = 0.000	P_KW_ = 0.000
	5.6 Pa–0.2 Pa	P_KW_ = 0.000	*p* = 0.000	P_KW_ = 0.000
	4.1 Pa–2.2 Pa	P_KW_ = 0.052	*p* = 0.000	P_KW_ = 0.058
	4.1 Pa–0.2 Pa	P_KW_ = 0.000	*p* = 0.000	P_KW_ = 0.000
	2.2 Pa–0.2 Pa	P_KW_ = 0.078	P = 1.000	P_KW_ = 0.277

*Note*: P = N indicates a result obtained using an ANOVA, P_KW_ = N indicates a result obtained using a Kruskal–Wallis test.

Abbreviations: ANOVA, analysis of variance; LDPE, low‐density polyethylene.

**Table A4:** *p* values from the statistical analysis comparing *Cobetia marina* results from each of the three surfaces, divided by shear

Shear (Pa)	Surfaces	Biomass	Maximum thickness	Surface area
**0.2**	Glass ‐ Permanox®	P_KW_ = 1.000	P_KW_ = 0.024	P_KW_ = 0.740
	Glass ‐ LDPE	P_KW_ = 0.036	P_KW_ = 0.000	P_KW_ = 0.000
	Permanox® ‐ LDPE	P_KW_ = 0.237	P_KW_ = 0.026	P_KW_ = 0.022
**2.2**	Glass ‐ Permanox®	P_KW_ = 0.751	P = 1.000	P_KW_ = 1.000
	Glass ‐ LDPE	P_KW_ = 0.002	*p* = 0.000	P_KW_ = 0.001
	Permanox® ‐ LDPE	P_KW_ = 0.061	*p* = 0.000	P_KW_ = 0.017
**4.1**	Glass ‐ Permanox®	P = 1.000	P = 1.000	P_KW_ = 1.000
	Glass ‐ LDPE	*p* = 0.999	*p* = 0.043	P_KW_ = 0.018
	Permanox® ‐ LDPE	*p* = 0.200	*p* = 0.124	P_KW_ = 0.074
**5.6**	Glass ‐ Permanox®	P = 1.000	*p* = 0.343	*p* = 0.690
	Glass ‐ LDPE	*p* = 0.048	P = 1.000	*p* = 0.211
	Permanox® ‐ LDPE	*p* = 0.078	*p* = 0.310	P = 1.000

*Note*: P = N indicates a result obtained using an ANOVA, P_KW_ = N indicates a result obtained using a Kruskal–Wallis test.

Abbreviations: ANOVA, analysis of variance; LDPE, low‐density polyethylene.

**Table A5:** *p* values from the statistical analysis comparing *Pseudomonas aeruginosa* results from each of the three surfaces, divided by shear

Shear (Pa)	Surfaces	Biomass	Maximum thickness	Surface area
**0.2**	Glass ‐ Permanox®	P_KW_ = 0.004	P_KW_ = 0.128	P_KW_ = 0.714
	Glass ‐ LDPE	P_KW_ = 0.762	P_KW_ = 0.004	P_KW_ = 0.998
	Permanox® ‐ LDPE	P_KW_ = 0.000	P_KW_ = 0.701	P_KW_ = 0.103
**2.2**	Glass ‐ Permanox®	P_KW_ = 0.394	*p* = 0.050	P_KW_ = 1.000
	Glass ‐ LDPE	P_KW_ = 0.031	*p* = 0.006	P_KW_ = 1.000
	Permanox® ‐ LDPE	P_KW_ = 0.000	P = 1.000	P_KW_ = 1.000
**4.1**	Glass ‐ Permanox®	*p* = 0.526	*p* = 0.209	P_KW_ = 0.766
	Glass ‐ LDPE	*p* = 0.000	P = 1.000	P_KW_ = 0.099
	Permanox® ‐ LDPE	*p* = 0.001	*p* = 0.558	P_KW_ = 0.915
**5.6**	Glass ‐ Permanox®	*p* = 0.054	*p* = 0.892	*p* = 0.037
	Glass ‐ LDPE	*p* = 0.000	*p* = 0.041	*p* = 0.000
	Permanox® ‐ LDPE	*p* = 0.014	*p* = 0.425	*p* = 0.385

*Note*: P = N indicates a result obtained using an ANOVA, P_KW_ = N indicates a result obtained using a Kruskal–Wallis test.

Abbreviations: ANOVA, analysis of variance; LDPE, low‐density polyethylene.
